# High blood pressure prevalence, awareness, treatment, and blood pressure control among Ugandans with rheumatic and musculoskeletal disorders

**DOI:** 10.1371/journal.pone.0289546

**Published:** 2023-08-07

**Authors:** Winnie Kibone, Felix Bongomin, Jerom Okot, Angel Lisa Nansubuga, Lincoln Abraham Tentena, Edbert Bagasha Nuwamanya, Titus Winyi, Whitney Balirwa, Sarah Kiguli, Joseph Baruch Baluku, Anthony Makhoba, Mark Kaddumukasa

**Affiliations:** 1 School of Medicine, College of Health Sciences, Makerere University, Kampala, Uganda; 2 Faculty of Medicine, Gulu University, Gulu, Uganda; 3 Department of Medicine, Kiruddu National Referral Hospital, Kampala, Uganda; 4 Department of Medicine, St. Francis Hospital Nsambya, Kampala, Uganda; Universal Scientific Education and Research Network, CAMEROON

## Abstract

**Background:**

Rheumatic and musculoskeletal disorders (RMDs) are associated with cardiovascular diseases (CVDs), with hypertension being the most common. We aimed to determine the prevalence of high blood pressure (HBP), awareness, treatment, and blood pressure control among patients with RMDs seen in a Rheumatology clinic in Uganda.

**Methods:**

We conducted a cross-sectional study at the Rheumatology Clinic of Mulago National Referral Hospital (MNRH), Kampala, Uganda. Socio-demographic, clinical characteristics and anthropometric data were collected. Multivariable logistic regression was performed using STATA 16 to determine factors associated with HBP in patients with RMDs.

**Results:**

A total of 100 participants were enrolled. Of these, majority were female (84%, n = 84) with mean age of 52.1 (standard deviation: 13.8) years and median body mass index of 28 kg/m^2^ (interquartile range (IQR): 24.8 kg/m^2^–32.9 kg/m^2^). The prevalence of HBP was 61% (n = 61, 95% CI: 51.5–70.5), with the majority (77%, n = 47, 95% CI: 66.5–87.6) being aware they had HTN. The prevalence of HTN was 47% (n = 47, 37.2–56.8), and none had it under control. Factors independently associated with HBP were age 46-55years (adjusted prevalence ratio (aPR): 2.5, 95% confidence interval (CI): 1.06–5.95), 56–65 years (aPR: 2.6, 95% CI: 1.09–6.15), >65 years (aPR: 2.5, 95% CI: 1.02–6.00), obesity (aPR: 3.7, 95% CI: 1.79–7.52), overweight (aPR: 2.7, 95% CI: 1.29–5.77).

**Conclusion:**

There was a high burden of HBP among people with RMDs in Uganda with poor blood pressure control, associated with high BMI and increasing age. There is a need for further assessment of the RMD specific drivers of HBP and meticulous follow up of patients with RMDs.

## Introduction

Hypertension (HTN) is a significant public health concern globally, with its prevalence continually rising in developing countries. According to the World Health Organisation, an estimated 1.28 billion adults aged 30–79 years worldwide have hypertension, with about two thirds living in low- and middle-income countries such as Uganda [1]. Moreover, nearly half (46%) are unaware that they have hypertension, 42% are diagnosed and treated, and only 21% have it under control [1]. In Uganda, the prevalence of hypertension varies with regions of the country, with the highest prevalence registered in the central region, estimated at about 34.3% and the lowest in Northern Uganda, estimated at 22.0% according to a national epidemiological study published in 2018 [2]. In addition, among private patients in the central region, the prevalence of hypertension was estimated at 41.6% and elevated blood pressure (BP) at 37.6%, but only 18.3% achieved control [3].

Patients with rheumatic and musculoskeletal diseases (RMDs) such as rheumatoid arthritis (RA), systemic lupus erythematous (SLE), osteoarthritis (OA), gout and Sjogren’s syndrome are at an increased risk of developing HTN, with chronic inflammation as the main driver [4–6]. Chronic inflammation causes damage to the blood vessels and contributes to salt retention in the body through the inflammatory cytokines such as interleukin-6 and tumor necrosis factor-alpha stimulating the renin-angiotensin-aldosterone system [7]. Other factors associated with the development of HTN among patients with RMDs include traditional cardiovascular risk factors such as genetic predisposition, advanced age, obesity, physical inactivity and use of nonsteroidal anti-inflammatory drugs and corticosteroids [8–10].

Anyfanti and colleagues, in a study conducted among patients with RMDs attending Rheumatology Outpatient Clinics in Greece, showed a high prevalence of HTN, estimated at 54.5%, with 21.7% being unaware that they had HTN [11]. In addition, patients with RMDs such as RA are at a 50% increased risk of cardiovascular related morbidity and mortality compared to the general population [12, 13]. However, HTN among most patients with RMDs is undiagnosed, and therefore poorly controlled, yet it is the main risk factor for developing cardiovascular events [14, 15]. Mandatory regular screening for HTN among all patients diagnosed with RMDs is a reliable means for early detection, thus early and effective management of the condition [16]. The burden of hypertension among patients with RMDs in Uganda remains unknown. However, many cases of hypertension go undiagnosed, and thus treatment is not availed, increasing mortality and morbidity in this population [17].

Therefore, in the present study, we aimed to determine the prevalence of high blood pressure (HBP), awareness, treatment, and BP control among patients with RMDs seen in a Rheumatology clinic in Uganda.

## Materials and methods

### Study design, setting & population

Using the Strengthening the Reporting of Observational Studies in Epidemiology (STROBE) guidelines, we conducted a cross-sectional analytical study. Data were collected prospectively between January and April 2022. The study was conducted in the Rheumatology Clinic of Mulago National Referral Hospital (MNRH), Kampala, Uganda. MNRH, located in the capital city, Kampala, is the largest public health facility in Uganda serving as a national specialized hospital with over 1800-bed capacity. The clinic is run by a rheumatologist (MK) assisted by other medical officers and registered nurses and has over 200 patients with various RMDs. The clinic is the largest in the country and serves as the national referral center for patients with RMDs across the country.

We included patients of Ugandan nationality aged 18 years and above, with a confirmed diagnosis of RMD, irrespective of the gender, attending the MNRH Rheumatology Clinic after having given the consent to participate. The RMDs of interest were: RA classified according to 2010 European League Against Rheumatism (EULAR)/ American College of Rheumatology (ACR) criteria [18], SLE classified according to the 2019 EULAR/ACR criteria [19], gout classified according to the 2015 EULAR/ACR criteria [20], Sjogren’s syndrome classified according to the 2016 EULAR/ACR criteria [21], OA classified according to the 1986, 1990 and 1991 EULAR/ACR criteria osteoarthritis of the knee, hand and hip respectively [22–24], spondyloarthropathies classified according to the 2011 ASAS criteria [25] and Anti-neutrophil cytoplasmic antibody (ANCA)-associated vasculitis classified according to the 2022 EULAR/ACR criteria [26]. We excluded patients who declined to participate or were unable to give informed consent.

### Data collection

Data classified into socio-demographic, clinical (RMD diagnosis, duration and treatment, BP readings, HBP and HTN awareness, HTN treatment and BP control) and anthropometric (weight, height, and waist-hip ratio) were collected using an interviewer administered semi structured questionnaire and direct measurements taken by the interviewers. The questionnaires were printed out and administered by the interviewers to the respondents at the Rheumatology clinic, to ensure that the respondents understand the questions. Three BP measurements were taken on the same day on two occasions, four hours apart, on both arms using a manual sphygmomanometer because it is the recommended method for accurately assessing BP [27]. Each participant was at the triage station for at least five minutes to allow them to relax and for their BP to stabilize to avoid falsely elevated readings. BP was taken when the participant was seated upright with his/her arm at the heart level in a quiet triage room to ensure accurate readings, and it was done by routine care nurses to reduce the likelihood of white coat hypertension. Office BP was determined as an average of the three readings.

Awareness was assessed by asking the patient whether they were hypertensive or not using a single question.

### Statistical analysis

The sample size of 194 participants with RMDs was calculated using the modified Kish and Leslie formula [28] for finite population size, with an estimated prevalence of HBP of 54.5% from a similar study conducted in Greece [11], a population size of 200, and type 1 error of 5%, and a standard deviation at 95% confidence interval (1.96). Adjusting for the finite population, using Slovin’s formula (N/(1+Ne^2^), where N = 150, and e is 5%, a final sample size of 130 participants was obtained [29].

Data was cleaned, coded and entered into Microsoft 2016, and exported for analysis using the STATA version 17.0 analysis software. Numerical data were assessed for normality using Shapiro-Wilk test. Continuous variables were expressed as mean and standard deviation for parametric variables or median and interquartile range for non-parametric variables. Categorical data were presented as frequencies and percentages. Chi-square or Fischer’s exact tests were used to compare categorical variables as appropriate and student t-tests or Mann-Whitney U for numerical variables. Modified Poisson regression was used to determine the factors associated with HBP and association was measured using the prevalence ratio because the prevalence of HBP is high, which may bias odds ratio values [30–32]. We conducted bivariate analysis and variables with p<0.20 were considered for multivariate analysis. In the multivariate analysis, we used manual backward elimination method until all the variables in the model had p-value ≤ 0.05. We assessed for interaction by forming product terms and performed a chunk test. We assessed for confounding by considering a percentage change of > 10% in the crude and adjusted prevalence ratios. The goodness of fit of the model was assessed using Hosmer-Lemeshow goodness of fit test. Variables with p< 0.05 were considered statistically significant.

### Operational definitions

HBP was defined as an office systolic blood pressure (SBP) ≥ 140mmHg and/or diastolic blood pressure (DBP) ≥90 mmHg [27]. HBP awareness, as knowledge of the participant about being hypertensive based on previous diagnosis by a qualified health care worker. HBP treatment was defined as a patient being on antihypertensive medicine. Hypertension, as a patient self-reporting to be hypertensive or ongoing antihypertensive drug treatment. Uncontrolled hypertension, as a patient with a known diagnosis of hypertension on antihypertensive medicine whose blood pressures are ≥140/90 on the day of the current clinic visit (day of the interview).

### Ethical consideration

Ethical approval was sought from Mulago Hospital Research and Ethics Committee (Approval number: MHREC 2162). Approved by MHREC, a modification was made to the initial protocol from targeting only patients with RA to include patients with RMDs. All participants provided written informed consent by signing a consent form appended to the questionnaire. The study was conducted in observance of the *Declaration of Helsinki-*2013.

## Results

**Fig 1:**
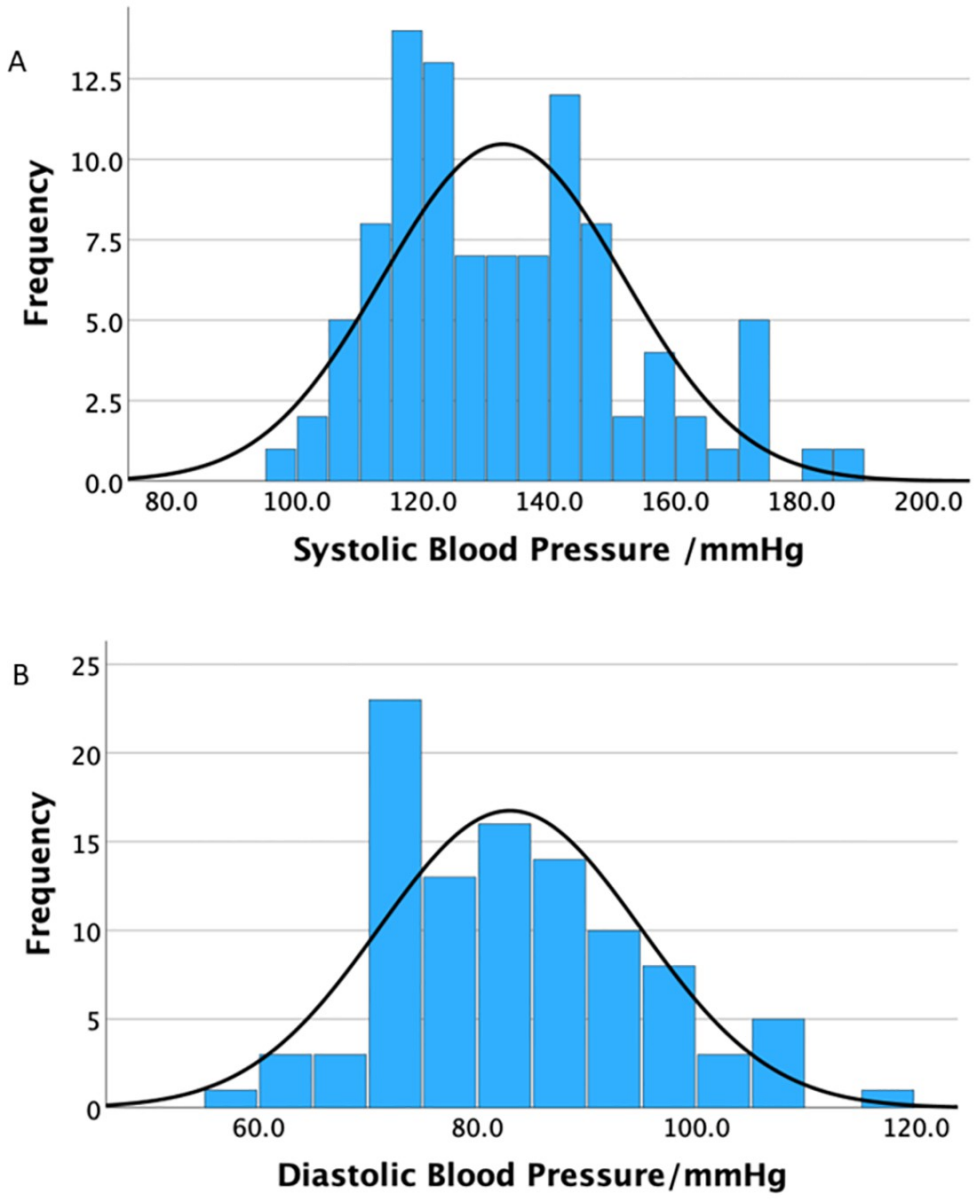
Graphs showing the trends of systolic and diastolic blood pressure in participants.

Of the anticipated 130 participants, 110 participants turned up at rheumatology clinic during the study period. However, only 100 participants met the eligibility criteria and were enrolled, resulting in a positive response rate of 100/110. No significant differences were observed between the participants and non-participants in terms of age, gender, or disease severity. The majority of participants were female (84%, n = 84), with mean age of 52.1(SD: 13.8) years and median BMI of 28 kg/m^2^ (IQR: 24.8 kg/m^2^-32.9 kg/m^2^). Fifty-nine participants were employed (59%), from urban residency (69%, n = 69), majority had no history of smoking (96%, n = 96) or use of alcohol (67%, n = 67) and forty-nine had family history of hypertension (49%). Eight (8%) participants had diabetes mellitus. Overall, the median SBP and DBP were 129.3(IQR: 118.3–144.7) mmHg and 81.3(IQR: 74–90.7) mmHg, respectively. The trends of systolic and diastolic blood pressure in participants are shown in Fig 1. Table 1 summarizes socio-demographic and clinical characteristics of all the study participants.

**Table 1:** Demographic and clinical characteristics of all the study participants stratified by presence or absence of high blood pressure.

Variable	All (n = 100) n(%)	HBP		p-value
		Yes (n = 61) n(%)	No (n = 39) n(%)	
**General characteristics of the study population**				
Age category, years	52.1 (13.8)	55.7(1.6)	46.0(2.3)	0.001
18–25	4(4.0)	1(1.6)	3(7.7)	0.015
26–35	10(10.0)	4(6.6)	6(15.4)	
36–45	14(14.0)	4(6.6)	10(25.6)	
46–55	32(32.0)	23(37.7)	9(23.1)	
56–65	23(23.0)	17(27.9)	6(15.4)	
>65	17(17.0)	12(19.7)	5(12.8)	
Sex				
Female	84(84.0)	52(85.3)	32(82.1)	0.671
Male	16(16.0)	9(14.7)	7(17.9)	
occupation				
Employed	59(59.0)	35(57.4)	24(61.5)	0.835
Unemployed	41(41.0)	26(42.6)	15(38.5)	
Residency				
Peri-urban	28(28.0)	15(24.6)	13(33.3)	0.270
Rural	3(3.0)	3(4.9)	0(0.0)	
Urban	68(69.0)	43(70.5)	26(66.7)	
Religion				
Anglican	31(31.0)	23(37.7)	8(20.5)	0.372
Born again	16(16.0)	9(14.8)	7(18.0)	
Catholic	32(32.0)	16(26.2)	16(41.0)	
Moslem	18(18.0)	11(18.0)	7(17.0)	
Seventh Day Adventist	3(3.0)	2(3.3)	1(2.6)	
Education				
No formal education	4(4.0)	3(4.9)	1(2.6)	0.694
Primary	31(31.0)	21(34.4)	10(25.6)	
Secondary	41(41.0)	24(39.3)	17(43.6)	
Tertiary	24(24.0)	13(21.3)	11(28.2)	
Marital status				
Divorced	13(13.0)	10(16.4)	3(7.7)	0.283
Married	46(46.0)	28(45.9)	18(46.2)	
Single	22(22.0)	10(16.4)	12(30.8)	
Widowed	19(19.0)	13(21.3)	6(15.4)	
Smoking status				
Former	4(4.0)	2(3.3)	2(5.1)	0.642
Never	96(96.0)	59(96.7)	37(94.9)	
Alcohol use				
Current	18(18.0)	12(19.7)	6(15.4)	0.717
Former	15(15.0)	10(16.4)	5(12.8)	
Never	67(67.0)	39(63.9)	28(71.8)	
Family history of hypertension				
No	51(51.0)	27(44.3)	24(61.5)	0.105
Yes	49(49.0)	34(55.7)	15(38.5)	
Family history of diabetes mellitus				
No	85(85.0)	52(85.3)	33(84.6)	>0.999
Yes	15(15.0)	9(14.7)	6(15.4)	
History of diabetes mellitus,	8(8.0)	8(13.1)	0(0.0)	0.020
RMD diagnosis				
Gout	5(5.0)	5(8.2)	0(0.0)	0.067
Osteoarthritis	11(11.0)	6(9.8)	5(12.8)	
Rheumatoid arthritis	79(79.0)	49(80.3)	30(76.9)	
Others (SLE, spondyloarthropathies, sjogren’s syndrome and ANCA-associated vasculitis)	5(5.0)	1(1.6)	4(10.3)	
Corticosteroid use	48 (48.0)	25 (41.0)	23 (59.0)	0.103
Body mass index, kg/m2, median (IQR)*	28(24.8–32.9)	30.9(27.2–35.3)	24.9(22.5–27.9)	<0.001
Normal weight	26(26.0)	6(9.8)	20(51.3)	<0.001
Obese	39(39.0)	33(54.1)	6(15.4)	
Overweight	35(35.0)	22(36.1)	13(33.3)	
Waist-Hip ratio*	0.90(0.8–0.9)	0.86(0.8–0.9)	0.80(0.8–0.9)	0.009

*Median with interquartile range (IQR)

The prevalence of HBP was 61% (n = 61, 95% CI: 51.5–70.5). Out of the 61 participants with HBP, the majority (77%, n = 47, 95% CI: 66.5–87.6) were aware they had HTN. The prevalence of HTN was 47% (n = 47, 95% CI: 37.2–56.8), and none had it under control. The most frequent RMDs were RA (80.3% in patients with HBP versus 76.9% in those without) followed by OA (9.8% in patients with HBP versus 12.8% in those without), gout (8.2% in patients with HBP versus 0% in those without) and other RMDs which are SLE, spondyloarthropathies, sjogren’s syndrome and ANCA-associated vasculitis (1.6% in patients with HBP versus 10.3% in those without). Individuals with HBP were older (p = 0.001) and had higher BMI (p<0.001) and median waist-to-hip ratio (0.86 vs 0.8, p = 0.009) compared to those without Table 1.

Factors independently associated with HBP were age 46-55years (adjusted prevalence ratio (aPR): 2.5, 95% CI: 1.06–5.95), 56–65 years (aPR: 2.6, 95% CI: 1.09–6.15), >65 years (aPR: 2.5, 95% CI: 1.02–6.00), obesity (aPR: 3.7, 95% CI: 1.79–7.52), and overweight (aPR: 2.7, 95% CI: 1.29–5.77) Table 2.

**Table 2:** Multivariable logistic regression analysis of predictors of hypertension among the study participants.

Variable	Adjusted prevalence ratio (95% Confidence Interval)
Age category, years	
18–25	0.9 (0.13–5.84)
26–35	1.4(0.45–4.33)
36–45	Reference
46–55	2.5 (1.06–5.95)
56–65	2.6 (1.09–6.15)
>65	2.5 (1.02–6.00)
Body mass index, kg/m^2^	
Normal weight	1.0 (Reference)
Obese	3.7 (1.79–7.52)
Overweight	2.7 (1.29–5.77)
Corticosteroid use	2.1 (1.02–7.12)

## Discussion

In this study, we aimed to determine the prevalence of HBP, awareness, treatment, and blood pressure control among patients with RMDs seen in a Rheumatology clinic in Uganda. However, comparability of our findings with data from the literature of RMDs is limited because, apart from one study conducted in Greece [11], this is the first study on HBP among patients with RMDs in Uganda and sub-Saharan Africa.

We found the prevalence of HBP to be 61%, with the majority (77%) of those aware that they had HTN, however, only 44.7% of participants had it under control. These findings align with results of the study conducted by Anyfanti and colleagues in Greece among patients with RMDs attending Rheumatology Outpatient Clinics [11]. The study reported a high prevalence of HTN among patients with RMDs, estimated at 54.5%, with 21.7% being unaware that they had HTN [11]. Similarly, a study by Protogerou and colleagues investigating HTN among patients with RA in Greece registered a high awareness level, estimated at about 80% [33]. As such, HTN is a common comorbidity in patients with RMDs, with high awareness of the condition attributed to factors such as increased awareness campaigns, improved healthcare access and effective communication between patients and health care providers [34, 35]. The limited BP control observed in our study is comparable to other studies conducted in Greece, which showed low BP control rates estimated at only 48.6% and 29% among patients with RMDs and RA respectively [11, 33]. Poor BP control in patients with RMDs is attributed to various factors, including poor patient adherence to treatment, healthcare provider awareness, and suboptimal therapeutic approaches such as inadequate medication titration [36, 37].

In our study, obesity was independently associated with HBP, consistent with previous studies conducted in both patients with RMDs and the general population [11, 38, 39]. Notably, we found that obesity exhibited a 1.5 times stronger association with HBP compared to overweight participants. The strong association between obesity and HBP underscores the importance of lifestyle modifications, including weight management, in the prevention and management of HTN in RMD patients. Obesity is closely linked to metabolic abnormalities and increased risk of cardiovascular diseases, including HTN [40]. Moreover, the burden of high BMI-related deaths and DALYs increases with age, especially in males, as evidenced by the 26.8% and 12.7% rise in the age-standardized rate of high-BMI-related DALYs for males and females, respectively, between 1990 and 2017 [41].

Our study revealed a significant association between increasing age from 36 years and HBP among participants. This finding could be explained by the higher proportion of older participants in our study. Aging is characterised by physiological changes in the cardiovascular system, including arteriosclerotic structural alterations and calcification, leading to large artery stiffness. Consequently, the blood vessels become less flexible and more rigid, resulting in elevated resistance to blood flow and higher BP levels. Moreover, aging is associated with chronic inflammation and oxidative stress, which, in conjunction with the chronic inflammation associated with RMDs further augments the risk of developing HBP [42]. Inflammation is known to be a major driver of HTN in patients with RMDs with elevated C reactive protein (CRP) an inflammatory marker and endothelial damage [5, 6]. Comparable to our findings, a recent study in China showed that risk of HBP increased from age 35 years in the general population [43]. In addition, Anyfanti and colleagues also reported a high prevalence of HTN among the elderly patients with RMDs [11]. Thus, our study underscores the role of age as a contributing factor to the development of HBP.

Rheumatologists, allied health professionals, and stakeholders involved in the care of patients with RMDs should consider incorporating lifestyle changes in patient care to reduce body fat, thereby preventing obesity [16, 44]. In addition, strategies such as health education and improved communication between healthcare providers and patients have the potential to empower patients with RMDs to be aware of their HTN status and make informed decisions regarding the condition, as well as to adopt healthy habits. It is crucial for elderly patients with RMDs in the rheumatology clinic to be closely monitored with more frequent blood pressure measurements to ensure early diagnosis and timely treatment of HTN.

Our study was not without limitations. Despite our study site being at a national referral hospital serving patients from across the country, it was a single centred study. Therefore, our findings are not generalizable to the Ugandan population. In addition, we used a non-standardized questionnaire which was however, developed based on expertise of physicians experienced in the management of RMDs in Uganda. We acknowledge that not measuring CRP levels among patients with RMDs limited our ability to directly investigate the potential link between inflammation in RMDs and HTN. Measuring CRP levels in future studies will be valuable in understanding the role of inflammation in the development and progression of HTN among patients with RMDs. In addition, we did not assess the causality of the associations observed because of cross-sectional study design. This was also the first study to assess the prevalence and factors associated with HBP in patients with RMDs in Uganda.

## Conclusion

This study shows a high burden of HBP among patients with RMDs in Uganda, with a considerable number being aware of their condition but experiencing poor BP control. The high burden was associated with high BMI and increasing age in this population. Thus, lifestyle modifications including weight management are crucial in preventing and managing HBP and its complications such as future cardiovascular disease and deaths. Continuous medical education and tailored guidelines for cardiovascular risk in RMDs are recommended.

## Supporting information

S1 Dataset (XLSX)
